# Programming of neuroendocrine self in the thymus and its defect in the development of neuroendocrine autoimmunity

**DOI:** 10.3389/fnins.2013.00187

**Published:** 2013-10-16

**Authors:** Vincent Geenen, Gwennaëlle Bodart, Séverine Henry, Hélène Michaux, Olivier Dardenne, Chantal Charlet-Renard, Henri Martens, Didier Hober

**Affiliations:** ^1^Department of Biomedical and Preclinical Sciences, Center of Immunoendocrinology, GIGA Research Institute, Fund of Scientific Research, University of LiegeLiege-Sart Tilman, Belgium; ^2^Laboratory of Virology EA3610, University of Lille 2 and CHRU LilleLille, France

**Keywords:** thymus, self-tolerance, autoimmunity, type 1 diabetes, neuroendocrine self-peptides, oxytocin, insulin-like growth factor 2, AIRE

## Abstract

For centuries after its first description by Galen, the thymus was considered as only a vestigial endocrine organ until the discovery in 1961 by Jacques FAP Miller of its essential role in the development of T (thymo-dependent) lymphocytes. A unique thymus first appeared in cartilaginous fishes some 500 million years ago, at the same time or shortly after the emergence of the adaptive (acquired) immune system. The thymus may be compared to a small brain or a computer highly specialized in the orchestration of central immunological self-tolerance. This was a necessity for the survival of species, given the potent evolutionary pressure imposed by the high risk of autotoxicity inherent in the stochastic generation of the diversity of immune cell receptors that characterize the adaptive immune response. A new paradigm of “neuroendocrine self-peptides” has been proposed, together with the definition of “neuroendocrine self.” Neuroendocrine self-peptides are secreted by thymic epithelial cells (TECs) not according to the classic model of neuroendocrine signaling, but are processed for presentation by, or in association with, the thymic major histocompatibility complex (MHC) proteins. The autoimmune regulator (AIRE) gene/protein controls the transcription of neuroendocrine genes in TECs. The presentation of self-peptides in the thymus is responsible for the clonal deletion of self-reactive T cells, which emerge during the random recombination of gene segments that encode variable parts of the T cell receptor for the antigen (TCR). At the same time, self-antigen presentation in the thymus generates regulatory T (Treg) cells that can inhibit, in the periphery, those self-reactive T cells that escaped negative selection in the thymus. Several arguments indicate that the origin of autoimmunity directed against neuroendocrine glands results primarily from a defect in the intrathymic programming of self-tolerance to neuroendocrine functions. This defect may be genetic or acquired, for example during an enteroviral infection. This novel knowledge of normal and pathologic functions of the thymus constitutes a solid basis for the development of a novel type of tolerogenic/negative self-vaccination against type 1 diabetes (T1D).

## Introduction

Galen (129–210 AC), who, with Hippocrates, is recognized as one of the fathers of Western medicine, first observed, behind the sternum and before the cardiac area, an organ that he named “thymus” because of its resemblance to the leaf of the thyme plant *Thymus vulgaris.* Galen also suspected that the thymus was the seat of courage and affection because of its close vicinity to the heart; in Ancient Greece, the word “thumos or thymos (θυμóς)” indicated a physical association between breath and blood, and referred to one of Plato's three constituent parts of human psyche.

The function of the thymus remained unknown for many centuries and it was considered only as a vestigial organ that had become redundant both during phylogeny and ontogeny after puberty. Then, at the beginning of the 20th century, Hammar (Sweden) published a series of important studies describing hyperplasia of the thymus in various endocrine diseases including acromegaly and Graves' disease, and after castration (Hammar, 1921). A few years later, Hans Selye reported that massive thymus atrophy is observed soon after administration of glucocorticoids (Selye, 1941), and this dramatic decrease in thymus size soon became a hallmark of the general adaptation and stress syndrome. At that time, the thymus was thought to be an endocrine gland but, despite the identification of several thymic “hormones,” the model of endocrine signaling failed to characterize the molecular dialogue between thymic epithelial cells (TECs) and immature thymic T cells (thymocytes). The hypothesis that the thymus had an endocrine nature then progressively faded, after Miller demonstrated the immunological function of the thymus (Miller, 1961). In this seminal paper, Miller postulated that lymphocytes leaving the thymus are specially selected in an epithelial environment. To the best of our knowledge, Burnet and Mackay published in *The Lancet* the first hypothesis connecting autoimmune diseases with histological abnormalities in the lympho-epithelial structure of the thymus (Burnet and Mackay, 1963). Immunological tolerance was first shown to be induced by thymic epithelial grafts in birds (Ohki et al., 1987), and the prominent role of the thymus in the induction of central tolerance was established by investigating the fate of developing thymocytes in response to superantigens (Kappler et al., 1987) or in T cell receptor (TCR) transgenic mice bearing a receptor for a HY-derived antigen (Kisielow et al., 1988). The identification of thymus-derived regulatory T cells (tTreg) (Sakagushi et al., 1995) completed our essential knowledge about the physiological function of the thymus, *i.e*., the generation of a diverse repertoire of TCRs that are self-tolerant and competent against non-self. The central role of thymus-dependent central self-tolerance has now become a cornerstone of immune physiology (Mathis and Benoist, 2004; Kyewski and Klein, 2006).

## Thymus organogenesis

Epithelial cells of the thymic cortex (cTECs, including thymic “nurse” cells) and medulla (mTECs) constitute around 85% of the thymus parenchyme. Other cells of the thymic stroma—dendritic cells (DCs) and macrophages—derive from the bone marrow. TECs arise from the endoderm of the 3rd pharyngeal pouch that, at around day 9 of embryonic development in the mouse (E9.0), contains thymic common epithelial progenitors (Blackburn and Manley, 2004; Anderson et al., 2007). Further development of this primitive epithelial rudiment between E9.5 and E12.5 depends on mesenchyme derived from the cephalic neural crest, the role of which extends beyond the early stages of thymus organogenesis (Foster et al., 2008).

Some human diseases and animal models involve a defect in thymus development, which leads to primary immune deficiencies. Di George's syndrome associates congenital absence or hypoplasia of the thymus and parathyroid glands together with defects in the heart and truncal vessels. This syndrome results partly from a migration failure of the cephalic neural crest. More recently, transgenic mouse models have revealed some of the molecular mechanisms involved in thymus development. Thus, *Hoxa3*^−/−^ mice present thymic aplasia, parathyroid hypoplasia, that are often accompanied by defects of the heart and the great truncal vessels (Manley and Capecchi, 1995). In mice, the “nude” phenotype results from mutations in the *nude* gene locus on chromosome 11 that encodes the transcription factor winged-helix nude (*whn*) or forkhead box N1 (*Foxn1*) (Nehls et al., 1994). *Foxn1* is a master regulator gene of the TEC differentiation programme; it controls lineage progression in cTECs and mTECs, and regulates a series of genes implicated in TEC function, although is not essential for medullary sublineage divergence (Nowell et al., 2011). Importantly, the genetic mechanisms responsible for murine thymus organogenesis are conserved in humans (Farley et al., 2013) and this is important with regard to current attempts to regenerate the thymus in aging, as well as in many other clinical conditions associated with compromised immune function (Holländer et al., 2010). Indeed, contrary to earlier dogma, the thymus is functional until an advanced age, and thymopoiesis plays a central role in immune reconstitution after intensive chemotherapy or anti-retroviral treatment (Douek et al., 1998).

## Overview of T cell differentiation in the thymus

The T cell immune system may be regarded as a 6th sensory organ that responds to different kinds of danger signals that are not detected by nerve cells. From the fetal liver, and later from the bone marrow, T lymphocyte progenitors migrate through the boundary between cortex and medulla into the thymic epithelial rudiment in response to chemoattractant factors (Wilkinson et al., 1999). They undergo many mitotic divisions in the outer cortex, and then differentiate after presentation of peptides by major histocompatibility complex (MHC) proteins expressed by thymic antigen-presenting cells (essentially cTECs, mTECs, DCs, and macrophages). The stochastic rearrangement of related β– then α-chains generates an enormous diversity of TCRs, many of which can bind self-antigen/MHC complexes with high affinity and are subsequently deleted (negative selection). Clonal deletion of thymocytes can occur both in the cortex and in the medulla (McCaughtry et al., 2008), but is most effective in the medulla.

Self-antigen presentation by thymic MHC proteins determines the whole process of T cell differentiation, which includes three alternative and exclusive fates for developing thymocytes: (1) Negative selection of self-reactive T cells generated during the random generation of TCR diversity due to the activity of the two recombination-activating enzymes RAG-1 and RAG-2; (2) Generation of self-specific tTreg cells; and (3) Positive selection of CD4+ and CD8+ effector and self-tolerant T cells. The first two events establish the establishment of the central arm of self-tolerance, while the avidity-affinity of the TCR—self-antigen—MHC interaction determines T cell negative or positive selection (Ashton-Rickardt et al., 1994). A remaining question is how the same MHC—self-peptide complexes can mediate either negative selection of self-reactive T cells or generation of self-specific tTreg cells (Klein et al., 2009; Stritesky et al., 2012). Another crucial question concerns the precise biochemical nature of self-peptides that are presented in the thymus, in particular during fetal life.

## Neurohypophysial family-related peptides and receptors in the thymus network

At the beginning of the 20th century, English scientists reported the galactogogue activity of thymus and corpus luteum extracts after their injection into the goat (Ott and Scott, 1910). At that time, oxytocin (OT) had not yet been identified as the specific factor of galactokinesis, which would be established in the late 50's by Vincent du Vigneaud and his team. In 1986, human thymus extracts were shown to possess potent uterine oxytocic activity, and OT was identified as the dominant member of the neurohypophysial family synthesized by TECs and thymic nurse cells in humans and different animal species (Geenen et al., 1986). Thymic T cells also express the OT receptor (OTR) and the V1b (or V3) vasopressin (AVP) receptor, which transduce nanomolar concentrations of OT and, accordingly to the rules of signaling induced by OTR and V1b, these receptors transduce low concentrations of OT and AVP via the phosphoinositide pathway in mitogenic signals for freshly isolated thymocytes (reviewed in Martens et al., 1996a, b). Together, these data showed the existence of a functional signaling within the thymus mediated by OT synthesized by TECs acting on functional OTR and V1b receptors expressed by developing T cells. Further molecular characterization of the neurohypophysial receptors present in the murine thymus showed that *Otr* is transcribed by all thymic T cell subsets, while *V1b* is expressed only in double positive CD4+8+ and single positive CD8+ T cells (Hansenne et al., 2005). Because intrathymic OT concentrations are in agreement with the high affinity Kd (10^−9^–10^−10^ M) of neurohypophysial receptors expressed by pre-T cells, the physico-chemical conditions are conducive to a functional signaling within the thymus network, which is not the case for the blood-borne OT and AVP, due to their low plasma concentration (10^−11^–10^−12^M).

Despite the strong evidence for the presence of neurohypophysial ligands and receptors in the thymus network, no secretion of OT or neurophysin could be detected in primary cultures of freshly isolated human TECs. Moreover, in the murine thymus, OT is not located in secretory granules but is diffuse in cytosol, in vesicles of the endoplasmic reticulum, and associated with keratin filaments (Wiemann and Ehret, 1993). This posed a fundamental problem, since the neuropeptide OT had been the basis for the development of the neurosecretion model by the Sharrer's in the 40′s. However, in 1990, Funder proposed a model of cell-to-cell cryptocrine (hidden secretion) signaling to characterize the direct membrane-to-membrane exchange of information between large epithelial nursing cells (like TECs in the thymus and Sertoli cells in the testis) and immature elements that migrate and differentiate at their contact (respectively, thymocytes and spermatocytes).

We thus proposed that, in the thymus, OT mediates a cryptocrine signaling between TECs and pre-T cells (Geenen et al., 1991). The observation of numerous points of focal adhesion between OT+ TECs and immature T cells (Wiemann and Ehret, 1993) prompted us to investigate the hypothesis that thymic OT could stimulate the activation of focal adhesion kinases (FAKs) in thymocytes. Among the proteins phosphorylated by OT in murine pre-T cells, two were precipitated with an anti-FAK mAb: one was identified as the p125^FAK^, while the other was a coprecipitating 130-kD protein (most probably p130^Cas^). In pre-T cells, OT also phosphorylates paxillin, a 68-kD protein located at focal adhesion sites and associated with p125^FAK^ (Martens et al., 1998). Together, these data establish the existence of a functional OT-mediated cryptocrine signaling in the thymus network. The OT-mediated promotion of focal adhesion may contribute to the establishment of immunological “synapses” between TECs and immature T cells, which is fundamental for the completion of the T cell differentiation programme.

## A paradigm shift: from thymic neuropeptides to “neuroendocrine self-peptides”

As discussed above, self-antigen presentation by thymic MHC proteins is the essential mechanism that determines the whole process of T cell differentiation. Because OT and its associated neurophysin are self-peptides synthesized in TECs, we hypothesized a processing of the thymic OT precursor that would be related to antigen presentation rather than classical neurosecretion. If true, such a processing would fit with the model of cryptocrine signaling, which implicates a membrane targeting of the ligand. Following affinity-chromatography with a mAb against the monomorphic part of human MHC class I molecules, we identified, in human TEC plasma membrane, a 55-kD protein that was labeled by both anti-MHC class I mAb and a specific anti-neurophysin antiserum. Because this antiserum does not cross-react either with MHC class I proteins, or with β2-microglobulin, this 55-kD membrane protein could represent a hybrid protein including a neurophysin domain (10 kD) and a MHC class I heavy chain-related domain (45 kD). The formation of this hybrid could reside at the post-transcriptional level (such as a trans-splicing event) or at the post-translational level (such as ATP-dependent ubiquitinylation). The MHC class I domain could be implicated in the membrane targeting of the hybrid, whereas the neurophysin domain would bind OT for final presentation to pre-T cells. According to this explanation, the neurophysin part of the OT precursor could fulfill the same function in the thymus and in the hypothalamo-neurohypophysial axis: binding of the peptide OT and transport to the external limit of TECs or magnocellular neurons, respectively. The tyrosine residue in position 2 of OT plays an important role in its binding to neurophysin (Griffin et al., 1973) and, interestingly, the tyrosine residue in the same position plays a crucial role in the binding of antigens to some MHC class I alleles for their presentation (Maryanski et al., 1991).

The antigenic behavior of thymic OT is also supported by another type of experiment. The recognition of OT by specific mAbs at the outer surface of TEC plasma membrane induces a marked increase in the secretion of interleukin 6 (IL-6) and leukemia inhibitory factor (LIF) in the supernatant of human TEC primary cultures (Martens et al., 1996a, b). Given the nature of the specific epitopes recognized by anti-OT mAbs, it was concluded that OT is fully processed at the level of the TEC plasma membrane. The absence of any effect of anti-AVP mAbs further supports the conclusion that thymic OT behaves as the self-peptide of the neurohypophysial family.

Because OT is a cyclic nonapeptide, and since antigen presentation mostly concerns linear sequences, the hypothesis of a similar processing was investigated with the linear neurotensin (NT). Primary cultures of human TECs contain around 5 ng of NT/10^6^ cells, of which 5% is associated with TEC plasma membranes. Again, no NT was detected in the supernatant of human TEC cultures. High performance liquid chromatography analysis of NT present in human TEC revealed a major peak of NT corresponding to intact NT_1−13_. Using the same affinity column prepared with an anti-MHC class I mAb, NT, and NT-related peptides were retained on the column and were eluted together with MHC class I proteins (Vanneste et al., 1997). The C-terminal sequence of NT includes tyrosine, isoleucine, and leucine residues that all can be used to anchor most of MHC class I alleles. Thus, NT and NT-derived C-terminal fragments could be natural ligands for MHC class I alleles.

At this point and in association with the characterization of the thymic peptides related to different neuroendocrine families (Geenen et al., 1998), the biochemical nature and properties of the *“neuroendocrine self”* could be defined according to the following features:

1. Neuroendocrine self-peptides are the dominant members of neuroendocrine gene/protein families that are expressed in TECs of many species and usually correspond to sequences that have been highly conserved during the evolution of a given family.
2. A hierarchy characterizes their expression profile. In the neurohypophysial family, OT is the dominant peptide expressed in TECs from different species. In the tachykinin family, neurokinin A (NKA)—but not substance P—is the peptide generated from the processing in TECs of the preprotachykinin A gene product (Ericsson et al., 1990). In the insulin family, all members are expressed in the thymus network: IGF-2 (cTECs and mTECs) > IGF-1 (cTECs, mTECs, and macrophages) >> Insulin (rare subsets of mTECs). This hierarchical pattern is important, because the strength of immunological tolerance to a protein/peptide is proportional to its concentration in the thymus (Ashton-Rickardt et al., 1994). Blocking thymic IGF-mediated signaling at the level of IGF ligands (in particular IGF-2) or IGF receptors interferes with the early stages of T cell differentiation, while one mAb to proinsulin did not exert any significant effect (Kecha et al., 2000).
3. The autoimmune regulator gene/protein (AIRE) controls the degree of intrathymic transcription of the genes encoding neuroendocrine self-peptides (Anderson et al., 2002).
4. In the thymus, neuroendocrine precursors are not processed according to the model of neurosecretion but undergo antigenic processing for presentation by—or in association with—MHC proteins. This processing differs between thymic antigen-presenting cells (APCs) and professional APCs (DCs, B cells, and macrophages) in the periphery. For some neuroendocrine self-peptides (OT and NT), such differences imply that their presentation in the thymus is not tightly restricted by MHC alleles as much as presentation of nonself antigens and autoantigens by dedicated APCs in the periphery.
5. For some precursors of neuroendocrine self-peptide precursors, their transcription in TECs precedes their orthotopic expression in peripheral neuroendocrine glands/cells (Hansenne et al., 2005).

Therefore, depending on their behavior either as the source of neuroendocrine self-peptides or cryptocrine signals, the thymic repertoire of neuroendocrine precursors recapitulates at the molecular level the dual role of the thymus in T cell negative and positive selection.

## Integrated coevolution of the neuroendocrine and immune systems (Figure 1)

Throughout evolution, the neuroendocrine and innate immune systems have evolved in parallel, and coexist in all animal species without any apparent aggression of the innate immune system toward neuroendocrine glands. Indeed, Toll-like receptors, which are the essential mediators of innate response, do not react against normal or undamaged self. Some anticipatory immune responses are present even in jawless vertebrates (agnathans), mediated by diverse variable lymphocyte receptors (VLRs), with 4–12 leucine-rich repeat modules assembled by some gene conversion process. Some 500 million years ago, the emergence of transposon-like genes coding for recombination-activating enzymes RAG-1 and RAG-2 in cartilaginous fishes (sharks and rays, mainly) initiated the development of adaptive immunity (Agrawal et al., 1998; Boehm and Bleul, 2007; Hirano et al., 2011). The appearance of these two genes in the genome of gnathostomes (probably via horizontal transmission), and the subsequent appearance of the combinatorial immune system has been sometimes described as the “Big Bang” of immunology. Gene recombination in somatic lymphoid cells is responsible for the stochastic generation of diverse immune receptors for antigen, B-cell receptors (BCRs, ± 5 × 10^13^ combinations) and T-cell receptors (TCRs, ± 10^18^ combinations). This extreme diversity of antigen receptors was directly associated with a high inherent risk of autoxicity that threatened survival of both species and individuals. This evolutionary pressure was so strong that, in accordance with Paul Ehrlich's concept of *horror autotoxicus*, novel structures, and mechanisms appeared with the specific role of protecting self against potential autoimmune attacks and orchestrating immunological self-tolerance. The first unique thymus also appeared in jawed cartilaginous fishes, concomitantly or very shortly after the emergence of adaptive immunity. The thymus did not abruptly appear but, as elegantly demonstrated by Bajoghli et al. (2011), was preceded by thymus-like lymphoepithelial structures in the gill baskets of lamprey larvae. These “thymoids” express the gene encoding forkhead box N4 (*Foxn4*), the ortholog of *Foxn1*, the transcription factor responsible for the differentiation of TECs in higher vertebrates as discussed above. *Foxn1* thus stands at a crucial place in the emergence of thymus epithelium that is essential for the control of T cell differentiation and self-tolerance programming (Boehm, 2011). The same study also provided strong evidence for a functional analogy between VLR assembly in thymoids and TCR recombination in the thymus, opening the hypothesis of autoimmune-like phenomena in jawless vertebrates.

**Figure 1:**
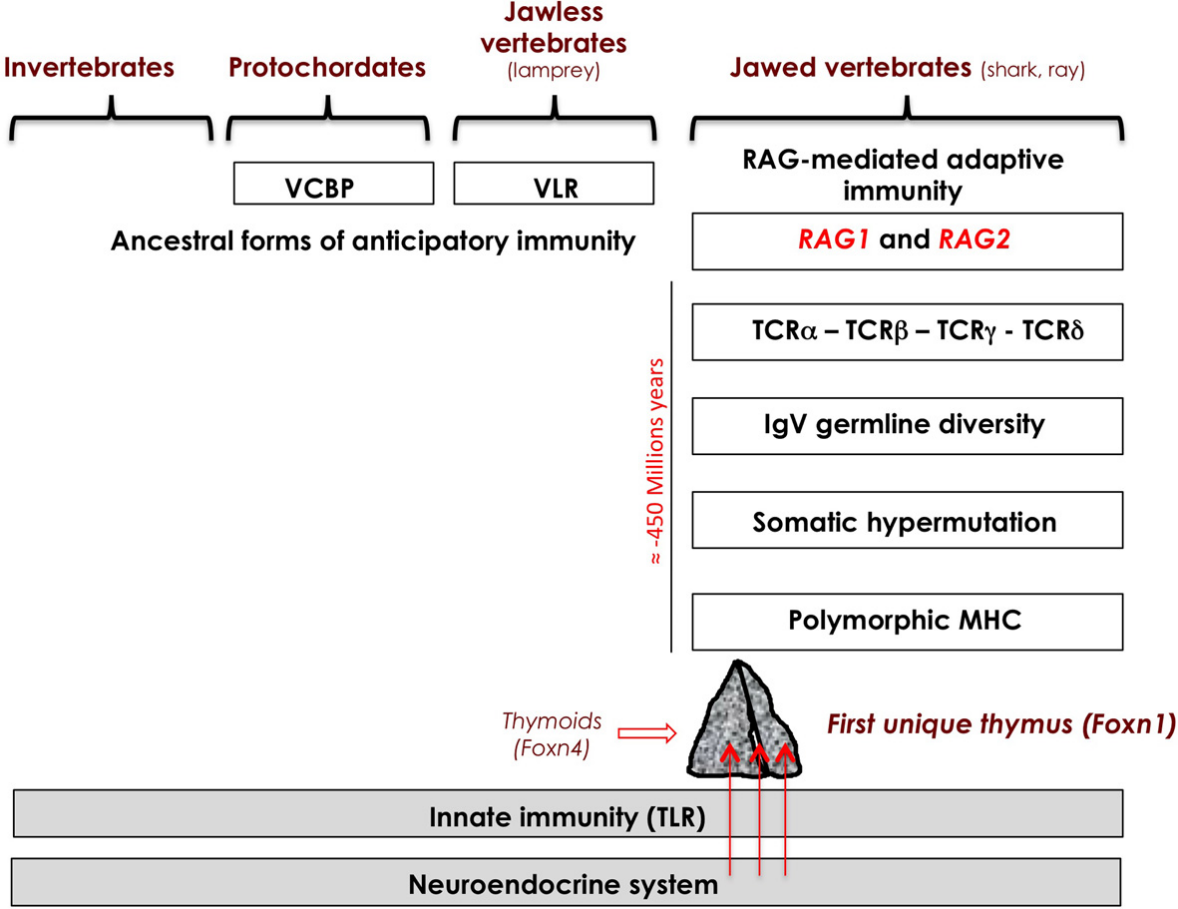
**Integrated evolution of the immune and neuroendocrine systems.** Neuroendocrine principles are evolutionarily ancient and did not evolve extensively except by gene duplication and differential RNA splicing. A high risk of inherent autoimmunity toward the neuroendocrine tissues resulted from the appearance of RAG-dependent adaptive immunity in jawed cartilaginous fishes. Preceded by ancestor thymoids in lamprey larvae, the first unique thymus emerged in jawed vertebrates, and the intrathymic presentation of neuroendocrine self-peptides (arrows) may be viewed *a posteriori* as a very efficient and economical way to instruct the adaptive T cell system in tolerating neuroendocrine functions as early as during thymus-dependent cell differentiation. VCBP, variable region-containing chitin-binding protein; VLR, variable lymphocyte receptor.

The hierarchic organization of the thymic repertoire of neuroendocrine self-peptides is also very significant from an evolutionary point of view. Because major neuroendocrine principles had been established before the emergence of the anticipatory adaptive immune response, they had to be protected from the risk of autoimmunity inherent to this immune lottery. OT is a hypothalamic nonapeptide that is closely implicated at different steps of the reproductive process, from social affiliation and bonding to control of parturition and lactation. Thus, OT is fundamental for the preservation of animal and human species. Through its dominant expression in TECs, OT is much more tolerated than AVP, its hypothalamo-neurohypohysial homolog, which mainly controls water homeostasis and vascular tone. Interestingly, rare cases of autoimmune hypothalamitis with AVP deficiency and diabetes insipidus have been repeatedly observed (de Bellis et al., 2004), but no autoimmunity against OT has ever been reported. Similarly in the insulin family, insulin is the primary autoantigen of type 1 diabetes (T1D) while there is no report of autoimmunity against IGF-2, a growth factor fundamental for fetus development and individual ontogeny. However, because of their close homology, however, thymic neuroendocrine self-peptides promote immunological cross-tolerance to their whole family, and tolerance to insulin is indeed lower in *Igf2*^−/−^ than in wild-type mice (Hansenne et al., 2006). Despite IGF-2 ubiquitous expression in extrathymic tissues, the deletion of *Igf2* in murine *Foxn1*+ TECs is also associated with a significant decrease in immunological tolerance to IGF-2 and to insulin (unpublished observations).

## A defect of central self-tolerance as the primary event driving development of autoimmunity (figure 2)

The evidence that T cells are programmed to recognize and tolerate the whole insulin family during their differentiation in the thymus prompted us to investigate the hypothesis of a defect in this education process as a potential source of self-reactive T cells directed specifically against insulin-secreting β cells of the pancreas. In other words, instead of considering autoimmune T1D as pathology of the pancreas, should we now consider the thymus as the primary defective organ? Already in 1973, Burnet theorized that the pathogenesis of autoimmune diseases first depends on a failure of self-tolerance and the appearance of “forbidden” self-reactive immune clones in the peripheral repertoire (Burnet, 1973), and even before the development of transgenic mice, a number of studies had provided elegant data supporting this hypothesis. Neonatal thymectomy prevents the emergence of autoimmune diabetes in an animal model of human T1D, the bio-breeding (BB) rat (Like et al., 1982). This benefit of neonatal thymectomy may now be explained by the removal of a defective thymus responsible for the continuous release and peripheral enrichment of the peripheral T cell repertoire with “forbidden” intolerant self-reactive T cells. Conversely, transplantation of thymus from diabetes-resistant (BBDR) to diabetes-prone BB (BBDR) rats prevents autoimmune diabetes in the latter (Georgiou and Bellgrau, 1989). Grafts of pure TECs from NOD mouse embryos to newborn C57BL/6 athymic nude mice also induce CD4 and CD8 T cell-mediated insulitis and sialitis (Thomas-Vaslin et al., 1997). Central tolerance and intrathymic apoptosis of self-reactive T cells were suspected to be defective in the NOD thymus (Kishimoto and Sprent, 2001; Zucchelli et al., 2005). However, a very careful recent study has established that thymic negative selection is functional in NOD mice (Mingueneau et al., 2012).

**Figure 2:**
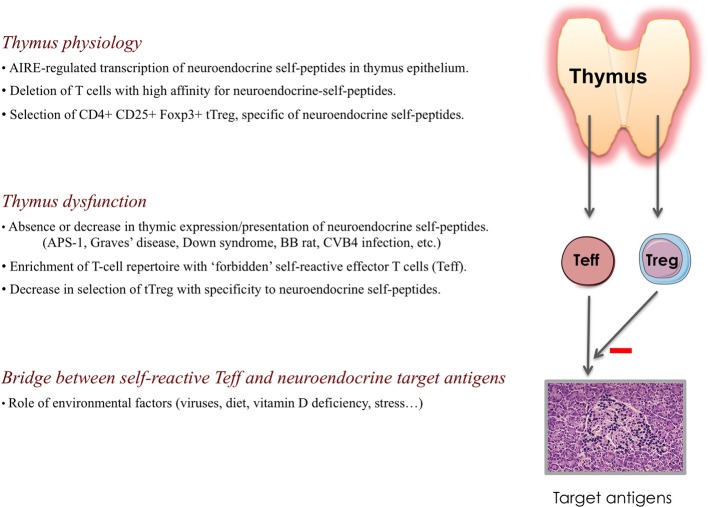
**Physiology of the thymus and the primary role of a thymus dysfunction in the development of autoimmune endocrine diseases.** Throughout life, the thymus generates naïve T cells that are self-tolerant and competent against nonself-antigens, as well as self-specific thymus-dependent regulatory T (tTreg) cells. Under AIRE control, thymus epithelium (and mTECs particularly) transcribes genes encoding neuroendocrine self-peptides, as well as other tissue-restricted antigens. The absence or decrease in the intrathymic presentation of neuroendocrine self-peptides conducts to a continuous enrichment of the peripheral T cell repertoire with “forbidden” self-reactive T cells (Teff) bearing a TCR directed against neuroendocrine self-antigens, while thymic generation of specific tTreg cells is severely impaired. This thymus defect results in the absence of central self-tolerance to neuroendocrine tissues/cells, which is a condition necessary but not sufficient to initiate an autoimmune endocrine disease. Environmental factors also intervene in the promotion of a molecular bridge between self-reactive Teff and target neuroendocrine antigens.

Transcription of all insulin-related genes has been investigated in the thymus of BBDR and BBDP rats. *Ins* and *Igf1* transcripts were detected in all BBDP and BBDR thymi whereas *Igf2* transcripts were also detected in all BBDR thymi but in only in 4 of 15 BBDP thymi. Such a defect of *Igf2* transcription in BBDP thymus could contribute both to their lymphopenia (including CD8+ T and RT6+ Treg cells) and to the absence of central tolerance to islet β cells (Kecha-Kamoun et al., 2001). In humans, *INS* transcripts are measured at a lower level in the thymus from fetuses with short class I variable number of tandem repeat (VNTR) alleles, the second genetic trait (*IDDM2*) of T1D susceptibility (Pugliese et al., 1997; Vafiadis et al., 1997). A number of other genetic loci associated with susceptibility to T1D could certainly determine disturbances in the thymus-dependent programming of central self-tolerance toward islet β cells.

In mice, where two genes code for (pro)insulin (*Ins1* and *Ins2*), *Ins2* is predominantly transcribed in rare subsets of mTECs while *Ins1* is dominantly expressed in murine islet β cells, which leads to a higher immunological tolerance to *Ins2*. This difference in the topography of *Ins2* and *Ins1* expression explains why breeding of *Ins2*^−/−^ mice onto the NOD background markedly accelerates insulitis and diabetes onset (Thebault-Baumont et al., 2003), while the incidence of insulitis and autoimmune diabetes is considerably reduced in *Ins1*^−/−^ congenic NOD mice (Moriyama et al., 2003). Susceptibility to autoimmune diabetes is also correlated with the level of *Ins2* transcription in the murine thymus (Chentoufi and Polychronakos, 2002). The role of thymic insulin in mediating central tolerance to islet β cells was demonstrated by the very rapid onset of autoimmune diabetes after a thymus-specific *Ins1* and *Ins2* deletion resulting from the crossing of *Ins1*^−/−^ mice with others presenting a specific *Ins2* deletion in *Aire*-expressing mTECs (Fan et al., 2009). Of note, *Ins2* transcription in mTEC clones is not regulated by glucose (Levi and Polychronakos, 2009) and is increased about 20-fold by anti-lymphotoxin β mAb (Palumbo et al., 2006). *Ins* transcription in mTECs uses a start site different than in pancreatic islet β cells (Villasenor et al., 2008). In addition to Aire, the insulin transactivator MafA also induces *Ins* transcription in the thymus; targeted *Mafa* disruption reduces *Ins2* expression in the thymus and induces anti-islet autoantibodies (Noso et al., 2010).

The identification of the autoimmune regulator (*AIRE*) gene, a member of the zinc-finger gene family, led to an extremely important advancement in our knowledge of the central role played by a thymus dysfunction in the pathogenesis of organ-specific autoimmune diseases (The Finnish-German APECED Consortium, 1997). *AIRE* mutations are responsible for a rare recessive congenital syndrome called autoimmune polyglandular syndrome type 1 (APS-1) or Autoimmune Poly-Endocrinopathy, Candidiasis, Ectodermal Dystrophy (APECED) syndrome. *AIRE* encodes a protein with structural characteristics of a transcription factor: its transcription is maximal in thymus epithelium, and *Aire*^−/−^ mice develop several autoimmune processes associated with a marked decrease in the intrathymic expression of numerous neuroendocrine self-peptides (Anderson et al., 2002). It also appears that both VNTR alleles (*IDDM2*) and the level of *AIRE* transcription in the thymus determine the concentration of *INS* transcripts in the human thymus (Sabater et al., 2005). The two plant homeodomains (PHDs) are critical regions for Aire function (Peterson et al., 2008); PHD2 strongly influences the ability of Aire to control the mTEC transcriptome and is therefore crucial for effective central tolerance induction (Yang et al., 2013). RANK signals from CD4+CD3− lymphoid tissue inducer (LTi) cells regulate the development and differentiation of *Aire*-expressing mTECs (Rossi et al., 2007). Very recently, a mutual interdependence between Aire and microRNAs (miRs) was evidenced in the thymus since the profile of miR expression is severely affected in isolated murine and human mTECs from *Aire*^−/−^ mice and since, in turn, *Aire* expression is downregulated in mTECs of Dicer null mutant mice (Macedo et al., 2012; Ucar et al., 2013).

An additional level of protection against autoimmunity is also provided by peripheral mechanisms of immunological tolerance that inactivate self-reactive T lymphocytes that have escaped the thymic censorship. Interestingly, extrathymic *Aire*-expressing cells were very recently identified as distinct bone marrow-derived tolerogenic cells that anergize effector self-reactive CD4+ T cells in secondary lymphoid organs (Gardner et al., 2008, 2013). These data further demonstrate that the Aire gene/protein is an essential shield against autoimmunity (Metzger and Anderson, 2011).

For several years, we have been investigating a novel hypothesis according which an infection by the enterovirus Coxsackie B4 (CVB4) could induce a thymus dysfunction and an impairment of central tolerance to the insulin protein/gene family. CVB4 can directly infect the epithelial and lymphoid compartments of the human and murine thymus, and promote a severe thymus dysfunction with massive pre-T cell depletion and a marked-up regulation of MHC class I expression by TECs and by double positive CD4+CD8+ immature thymic T cells (Brilot et al., 2002, 2004). CVB4 infection of murine fetal thymic organ cultures also interferes with T cell differentiation (Brilot et al., 2008). Outbred mice can be infected with CVB4 following oral inoculation, which results in systemic spreading of viral RNA and a prolonged detection of CVB4 RNA in thymus, spleen, and blood up to 70 days after inoculation (Jaïdane et al., 2006). Moreover, CVB4 infection of a murine mTEC line induces a dramatic decrease in *Igf2* mRNA and IGF-2 protein in this cell line, while *Igf1* transcripts were relatively unaffected. In this mTEC line, *Ins2* transcripts could not be detected (Jaïdane et al., 2012). Together, our data strongly suggest that CVB4 infection of the thymus could disrupt central self-tolerance to the insulin family, and could also enhance CVB4 virulence through induction of central tolerance to this virus.

With regard to autoimmune thyroiditis, which is the most frequent autoimmune endocrine disease, all major thyroid-specific antigens, i.e., thyroperoxydase, thyroglobulin, and thyrotropin receptor (TSHR), are also transcribed in human TECs in normal conditions (Paschke and Geenen, 1995; Sospedra et al., 1998). As first reported by Hammar, thymic hyperplasia is commonly observed in Graves' disease (Murakami et al., 1996), and homozygotes for an SNP allele predisposing to Graves' disease have significantly lower intrathymic *TSHR* transcripts than carriers of the protective allele (Colobran et al., 2011).

With regard to autoimmunity directed against peripheral nonendocrine organs, a defect in α-myosin expression in TECs was recently shown to exert a central role in the pathogenesis of autoimmune myocarditis in mice and humans (Lv et al., 2011; Lv and Lipes, 2012). Also, a defect in Aire-mediated central tolerance to myelin protein zero promotes the development of an autoimmune Th1 effector response toward peripheral nerves (Su et al., 2012).

## The concept of “negative/tolerogenic self-vaccination”

Although *Ins2* is expressed at low levels in rare mTECs, thymic (pro)insulin has an essential role in protecting islet β cells against diabetogenic autoimmunity. However, insulin *per se* does not exert any significant tolerogenic properties that could be exploited to reprogram immunological tolerance to islet β cells. With the exception of two studies (Fourlanos et al., 2011; Roep et al., 2013), all the clinical trials based on insulin administration by different ways failed to preserve the residual β cell mass once the autoimmune attack has induced patent T1D. On the contrary, the potent immunogenic properties of insulin were revealed in different studies (Blanas et al., 1996; Liu et al., 2002), and this immunogenicity could be related to the very low level of insulin gene transcription in the thymus. Other studies have also evidenced the risk of hypersensitivity or anaphylaxis following administration of an autoantigen (Pedotti et al., 2001).

Nevertheless, the development of peptide-based therapeutic vaccines remains an attractive approach because of the specificity of immune suppression or regulation directed to specific pathogenic self-reactive T cells. In this context, infusion of a strongly active insulin mimetope was recently shown to convert naïve T cells into Foxp3+ Treg cells *in vivo* and to prevent autoimmune diabetes in NOD mice (Daniel et al., 2011). We proposed that IGF-2 could be a safer and more valuable basis for developing a specific “negative/tolerogenic self-vaccination,” on the basis that *Igf2* transcription is defective in the thymus of BBDP rats (Kecha-Kamoun et al., 2001) and that IGF-2 mediates significant cross-tolerance to insulin (Hansenne et al., 2006). The concept of negative self-vaccination implies both the competition between IGF-2 and insulin-derived epitopes for presentation by DQ2 and DQ8 alleles, as well as a tolerogenic response—including recruitment of Treg cells–induced by MHC-presentation of IGF-2 self-peptides (Geenen et al., 2004, 2010).

## Conclusion

Our studies have established that the thymus is not a classical neuroendocrine gland, but an obligatory intersection between the adaptive immune and neuroendocrine systems (Geenen, 2010). Moreover, they have elucidated how thymic epithelium is responsible for the programming of immunological central self-tolerance toward neuroendocrine functions through presentation of neuroendocrine self-peptides. They have also helped to resolve three major questions concerning the pathogenesis of autoimmune endocrine disorders: Why are the neuroendocrine glands so frequently tackled by an autoimmune process? What is the origin of the pathogenic effector self-reactive T cells? And what explains the tissue/cell-specificity of the autoimmune processes? There is no doubt that this novel knowledge will soon be exploited for the development of innovative strategies to prevent and cure a variety of organ-specific devastating autoimmune diseases such as T1D.

### Conflict of interest statement

The authors declare that the research was conducted in the absence of any commercial or financial relationships that could be construed as a potential conflict of interest.
